# Intra-nanogap controllable Au plates as efficient, robust, and reproducible surface-enhanced Raman scattering-active platforms†

**DOI:** 10.1039/c9ra01813a

**Published:** 2019-04-29

**Authors:** Siyeong Yang, Minjin Kim, Sanghyeok Park, Hongki Kim, Jinyoung Jeong, Juyeon Jung, Eun-Kyung Lim, Min-Kyo Seo, Bongsoo Kim, Taejoon Kang

**Affiliations:** Department of Chemistry, KAIST 291 Daehak-ro, Yuseong-gu Daejeon 34141 Korea bongsoo@kaist.ac.kr; Department of Physics, KAIST 291 Daehak-ro, Yuseong-gu Daejeon 34141 Korea; Bionanotechnology Research Center, KRIBB 125 Gwahak-ro, Yuseong-gu Daejeon 34141 Korea kangtaejoon@kribb.re.kr; Department of Nanobiotechnology, KRIBB School of Biotechnology, UST 217 Gajeong-ro, Yuseong-gu Daejeon 34113 Korea; Environmental Disease Research Center, KRIBB 125 Gwahak-ro, Yuseong-gu Daejeon 34141 Korea

## Abstract

Practical application of surface-enhanced Raman scattering (SERS)-active platforms requires that they provide highly uniform and reproducible SERS signals. Moreover, to achieve highly stable and consistent SERS signals, it is important to control the nanostructured gaps of SERS-active platforms precisely. Herein, we report the synthesis of gap-controllable nanoporous plates and their application to efficient, robust, uniform, and reproducible SERS-active platforms. To prepare well-defined nanoporous plates, ultraflat, ultraclean, and single-crystalline Au nanoplates were employed. The Au nanoplates were transformed to AuAg alloy nanoplates by reacting with AgI in the vapor phase. The Ag in the alloy nanoplates was then chemically etched, thus forming well-defined SERS-active nanoporous plates. For the precise control of gaps in the nanoporous plates, we investigated the alloy forming mechanism based on X-ray photoelectron spectroscopy and transmission electron microscopy analyses. According to the mechanism, the composition of Ag was tunable by varying the reaction temperature, thus making the nanostructured gaps of the porous plates adjustable. We optimized the nanoporous plates to exhibit the strongest SERS signals as well as excellent uniformity and reproducibility. The computational simulation also supports the experimental SERS signals of nanoporous plates. Furthermore, we successfully performed label-free detection of a biocide mixture (5-chloro-2-methyl-4-isothiazolin-3-one/2-methyl-4-isothiazol-3-one) up to 10 ppm using Au nanoporous plates. The adoption of single-crystalline Au nanoplates, the novel synthesis method for alloy nanoplates in the vapor phase, and the investigation of alloy forming mechanisms synergistically contributed to the formation of well-defined nanoporous plates. We anticipate that the nanoporous plates will be useful for the practical sensing of trace chemical and biological analytes.

## Abstract 2

A gold plate with uniform and controllable intra-nanogaps was synthesized by the chemical vapor reaction of AgI vapor and Au nanoplate and subsequent etching and the nanoporous plate exhibited excellent SERS activity.

## Graphical abstract

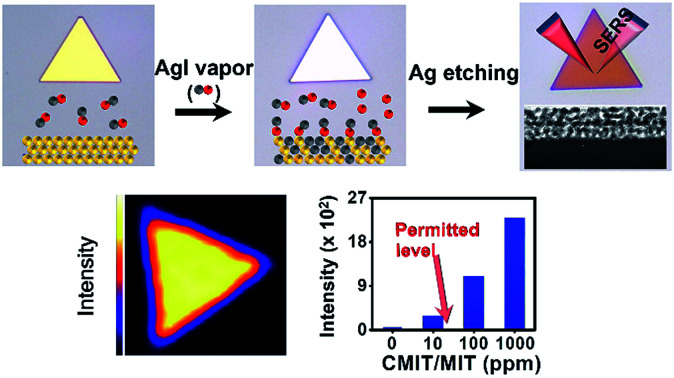

## Introduction

Surface-enhanced Raman scattering (SERS) is a fascinating phenomenon that can enhance the Raman signals of molecules up to 10^6^–10^14^.^1–3^ This remarkable enhancement is mainly attributed to hot spots, in which the electromagnetic field strongly increases.^4^ Although SERS has advantages such as single molecule-level sensitivity and molecular fingerprint spectrum, uncontrollable hot spots often cause a wide signal distribution, high variability, and uncertainty.^5–7^ Previous literature has suggested that only ∼0.1% of hot spots exhibited enhancement factor (EF) values larger than 1.0 × 10^8^.^8^ Therefore, it is critical to precisely control hot spots to obtain strong, stable, and consistent SERS signals. To date, various nanomaterials containing intra- or inter-nanogaps have been synthesized in the solution phase to adjust the size and number of hot spots. For example, nanostar, nanodumbell, nanoflower, and nanoleaf structures with intra-nanogaps were developed.^9–12^ In addition, assembled nanoparticle (NP) structures containing inter-nanogaps were reported.^13^ However, spontaneous aggregation of nanomaterials can induce ill-defined hot spots, lowering the uniformity and reproducibility of SERS signals.^14^ Moreover, the remaining ligand molecules could inhibit the sensitive and label-free sensing of analytes.^15,16^

Recently, porous nanostructures have been attracting attention as promising SERS-based sensing platforms because they have a large surface-to-volume ratio, thus having many hot spots and molecular binding sites.^6,16^ Accordingly, several methods, including de-alloying, galvanic replacement, and self-organization, have been reported to prepare porous structures.^17–22^ For instance, colloidal porous materials such as porous disks, porous ribbons, and porous nanospheres were synthesized.^16,23,24^ In addition, porous SERS-active substrates such as mesoporous Au films and wrinkled Au films were developed.^6,25^ It is still challenging, however, to precisely control the nanostructured gaps of porous structures. In this regard, we expected that the ultraflat, ultraclean, and single-crystalline Au nanostructures might be useful for the preparation of uniform and reproducible SERS-active porous nanostructures. Because the use of single-crystalline Au nanostructures has been advantageous for the construction of high-quality plasmonic nanostructures,^24,26,27^ the synthesis of well-defined porous nanostructures would be feasible by preserving the flatness, clearness, and crystallinity of the Au nanostructures.

Herein, we report a vapor phase synthesis of a well-defined nanoporous Au plate that provides excellent SERS enhancement, superb uniformity, and high reproducibility. The ultraflat, ultraclean, and single-crystalline Au nanoplates were employed as starting nanomaterials.^28^ The Au nanoplates were transformed to single-crystalline AuAg alloy nanoplates after reaction with AgI in the vapor phase. We investigated the alloy formation mechanism and thus can control the composition of alloy nanoplates accurately. After the chemical etching of the alloy nanoplates, well-defined porous nanoplates were obtained. These porous nanoplates have clean surfaces without any ligand molecules. More importantly, we carefully optimized the nanostructured gaps of the porous nanoplates and found that the porous nanoplates with an average gap size of 5.01 nm showed the maximum SERS enhancement. Theoretical calculations also showed the electric field enhancements of nanoporous plates corresponding to the experimental results. Furthermore, the Raman mapping suggested that an optimized nanoporous plate provided uniform SERS signals through the entire plate. The relative standard deviation (RSD) value of SERS signals measured from a single nanoporous plate was 4.7%, and the RSD value of signals from 18 nanoporous plates was 5.9%. Lastly, we detected label-free 5-chloro-2-methyl-4-isothiazolin-3-one/2-methyl-4-isothiazol-3-one (CMIT/MIT), a toxic chemical used in preservatives and disinfectants,^29^ up to 10 ppm by employing the Au nanoporous plate. Based on these results, we anticipate that the present porous nanoplates can be practical SERS substrates for the label-free detection of several chemicals and biomolecules with high reproducibility and sensitivity.

## Results and discussion

### Preparation of nanoporous plates

**Scheme 1:**
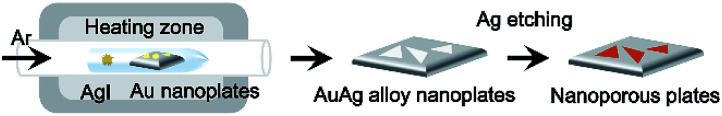
Schematic illustration of the synthetic procedure for nanoporous Au plates. Au nanoplates and AgI powder are placed in the glass reaction tube in the horizontal quartz tube furnace system. After the reaction in the furnace system, AuAg alloy nanoplates are synthesized. Next, the AuAg alloy nanoplates are selectively etched, and finally, the nanoporous Au plates are obtained.

Scheme 1 shows the synthetic procedures of the nanoporous Au plates. As starting materials, single-crystalline Au nanoplates are prepared according to a previous report.^28^ The prepared Au nanoplates and AgI powder are placed in a glass reaction tube, and then the reaction tube is put in the horizontal quartz tube furnace system. Next, the furnace system is heated under a flow of Ar gas at a rate of 150 standard cubic centimeters per minute (sccm). The pressure of the quartz tube is maintained at 0.7 torr during the reaction time of 40 min. As the temperature increases, AgI vaporizes and reacts with the Au nanoplates in the glass reaction tube. Because one side of the glass reaction tube is blocked, the vapor concentration of AgI in the reaction tube becomes high, enabling the effective formation of the AuAg alloy. When the Au nanoplates and AgI powder are put in the quartz tube without the glass reaction tube, the synthesis of AuAg alloy nanoplates is uncontrollable. After the formation of AuAg alloy nanoplates, the Ag in the alloy nanoplate is selectively etched by immersing the plates into nitric acid for 2 min. Finally, the nanoporous Au plate is obtained after washing.

**Fig. 1:**
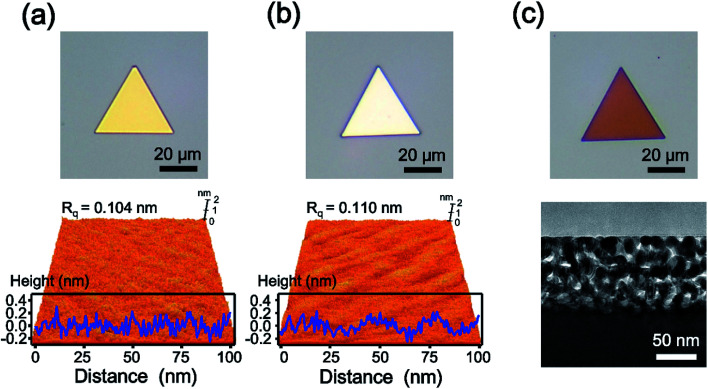
(a) Optical (top) and AFM (bottom) images of Au nanoparticles. The surface-height variation of the Au nanoplate is less than 0.5 nm with 0.104 nm of *R*_q_. (b) Optical (top) and AFM (bottom) images of the AuAg alloy nanoplate. The surface-height variation of the Au nanoplate is less than 0.6 nm with 0.110 nm of *R*_q_. (c) Optical (top) and cross-sectional TEM (bottom) images of nanoporous Au plates.

Fig. 1a shows the optical (top panel) and atomic force microscopy (AFM, bottom panel) images of a single-crystalline Au nanoplate. The optical micrograph displays the triangular shaped and gold-colored nanoplate. The 3-dimensional (3D) topographic image of AFM reveals the atomically smooth surface of the Au nanoplate. The surface-height variation of the Au nanoplate measured along the randomly selected line of 100 nm is less than 0.5 nm with 0.104 nm of root-mean-square roughness (*R*_q_). Considering that the atomic radius of Au is approximately 0.14 nm,^30^ these experimental values are noteworthy and indicate the atomically flat surface of the Au nanoplate. The cross-sectional transmission electron microscope (TEM) image of the Au nanoplate also shows the smooth surface of the nanoplate (Fig. S1†). Fig. 1b is the optical (top panel) and AFM (bottom panel) images of the AuAg alloy nanoplate. The optical image shows the color change of the nanoplate from gold to silver, indicating the successful transformation of the Au nanoplate to a AuAg alloy nanoplate. Interestingly, the AFM result suggests that the flatness of the Au nanoplate is retained after alloy formation. The surface-height variation in the alloy nanoplate is less than 0.6 nm with 0.110 nm of *R*_q_. These values are almost the same as those of the Au nanoplate, indicating that the AgI reacts with Au nanoplates while preserving the atomic flatness of nanoplates. During the reaction, the AgI molecules might be deposited onto a Au nanoplate without the formation of any cluster, and the Ag atoms might be homogeneously diffused into the Au nanoplate. After chemical etching of the AuAg alloy nanoplate, we obtained an optical image of the nanoporous Au plate (top panel of Fig. 1c). The image represents the triangular shaped and reddish colored plate. The cross-sectional TEM image of the porous nanoplate shows that the nanostructured gaps are formed densely and uniformly on the surface of the nanoplate (bottom panel of Fig. 1c). Moreover, the high-resolution TEM (HR-TEM) image shows that the crystallinity is preserved even in the porous nanoplate (Fig. S2†). From the optical, AFM, and TEM images, it is clearly verified that the nanoporous Au plates could be constructed from the single-crystalline Au plates.

### Alloying mechanism

**Fig. 2:**
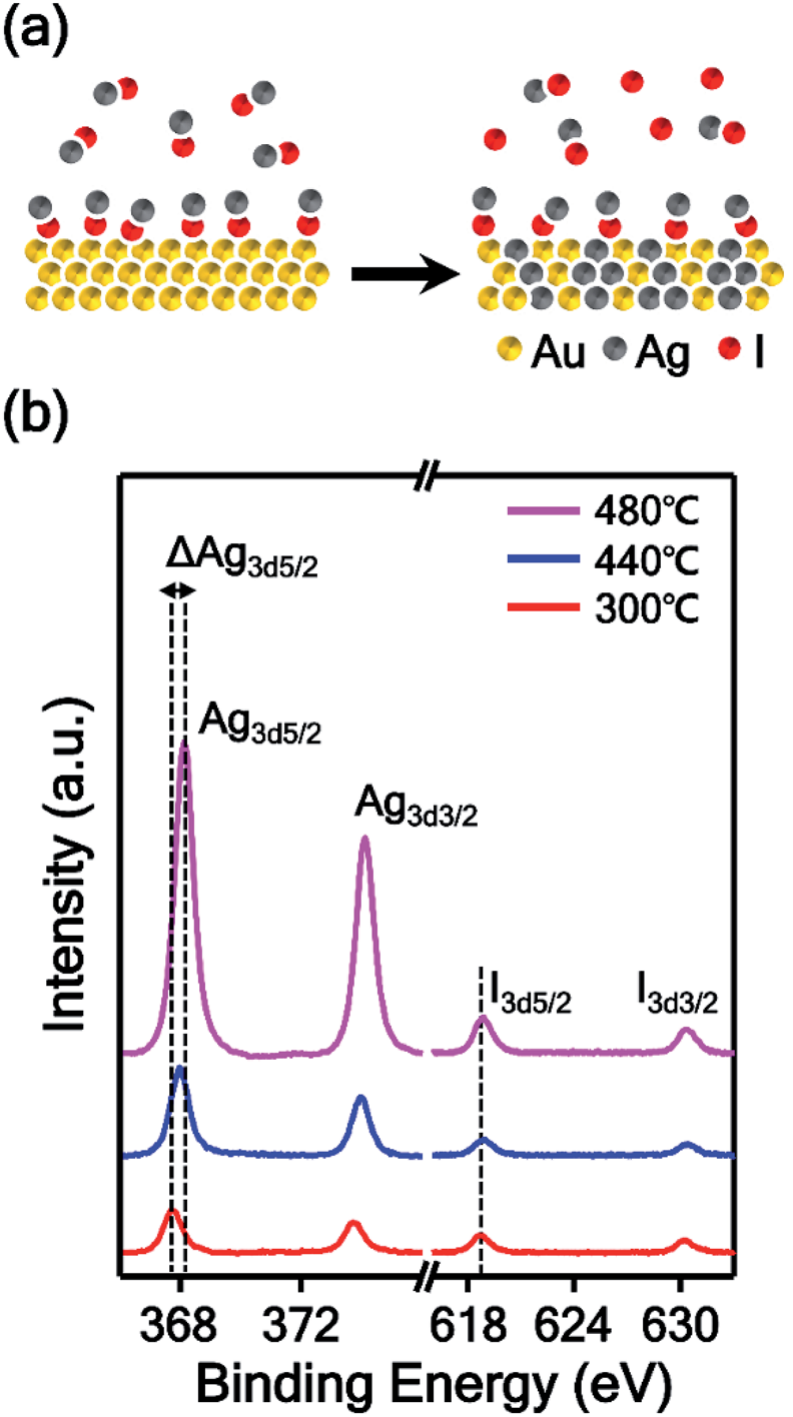
(a) Schematic illustration of the AuAg alloying process. The vaporized AgI is transported onto the Au nanoplate and binds with the surface Au atom through Au–I bonding. Then, the chemically adsorbed AgI decomposes to Ag and I, and finally, an AuAg alloy was formed. (b) XPS spectra of AuAg alloy nanoplates synthesized at temperatures of 300 (red spectrum), 440 (blue spectrum), and 480 °C (magenta spectrum).

In this experiment, AgI powder was employed as a precursor for the synthesis of AuAg alloy nanoplates. Therefore, we estimated the mechanism of the alloying process based on the thermodynamic data of AgI vapor. AgI decomposes into metallic Ag and elemental iodine over the temperature range of 250–1200 °C;^31,32^ thus, the AuAg nanoplates can be synthesized at the reaction temperature of 400 °C. At this reaction temperature range, Au atoms cannot vaporize actively, however, Au and Ag atoms can be mixed.^33^ We expect that Au atoms were preserved and no substitution occurred during the alloying reaction. Additionally, the thickness of nanoplates increased after the alloying reaction. When the reaction chamber is heated, AgI is vaporized and transported to the Au nanoplate by Ar carrier gas. Next, the transported AgI interacts with the surface Au atom and forms the Au–I bond, as shown in Fig. 2a. Previous studies showed that AgI can interact with Au by Au–I bonding.^34,35^ As the temperature increases, the chemically adsorbed AgI begins to decompose to Ag and I; subsequently, the Ag atoms start to form alloys with Au nanoplates. Because Au and Ag have the same lattice structures and very similar atomic radii, the alloy can be formed easily.^36,37^ In this synthetic method, the higher the reaction temperature is, the more Ag atoms are produced from AgI, and the further the Ag atoms diffuse into the Au nanoplate. Consequently, the alloy region could be expanded deeply into the inside of the plate as the reaction temperature increases. To prove the proposed mechanism, we performed X-ray photoelectron spectroscopy (XPS) of the alloy nanoplates synthesized under three different temperature conditions (300, 440, and 480 °C). The binding energies were calibrated with C1s (284.6 eV).^38–40^ As shown in Fig. 2b, the XPS spectra show interesting peak shift and intensity changes with the reaction temperature. First, the binding energies of Ag_3d5/2_ were measured at 367.7, 367.9, and 368.1 eV, respectively, from the alloy nanoplates synthesized at 300, 440, and 480 °C. Because the binding energy value of 367.7 eV corresponds to the value of Ag^+^,^41–43^ the XPS peak at 367.7 eV (red spectrum in Fig. 2b) indicates that the AgI was mainly present on the nanoplate synthesized at the low temperature of 300 °C.^42,43^ The binding energy value of 368.1 eV corresponds to the value of Ag^0^. Therefore, the shifted binding energy of Ag_3d5/2_ (ΔAg_3d5/2_ in Fig. 2b) suggests that AgI was decomposed at high temperatures (440 and 480 °C) and that the AuAg alloy was formed. Second, the binding energies of I_3d5/2_ were constant for all temperature conditions, but the peak intensities slightly increased with increasing reaction temperature. Since the iodine gas, generated from the decomposition of AgI, was carried away by the carrier gas, the presence of the I_3d5/2_ XPS peak indicates the presence of AgI on the nanoplates. This result implies that the vaporized AgI was continuously transported on the nanoplates through the whole reaction. Additionally, we found that the intensity ratio of Ag_3d5/2_ to I_3d5/2_ increased to 2.4, 5.6, and 8.7, depending on the reaction temperature. This result confirms that AgI was first transported on the nanoplates and decomposed to Ag and I; finally, an AuAg alloy was formed. Based on the mechanism, we could control the amount of Ag diffused into the Au nanoplate by adjusting the reaction temperature. Moreover, very weak XPS spectra were obtained from the substrate without nanoplates (Fig. S3†), suggesting the selective adsorption of AgI on the Au nanoplates during the reaction. The selective adsorption of AgI on the Au nanoplates and subsequent decomposition of AgI enabled the effective synthesis of AuAg alloy NPs in the vapor phase at lower temperatures than the previous vapor phase alloy synthesis method using a metallic Ag source.^44^ To the best of our knowledge, this is the first demonstration of the selective adsorption of vaporized AgI onto the Au surface and the subsequent formation of the AuAg alloy in the vapor phase.

**Fig. 3:**
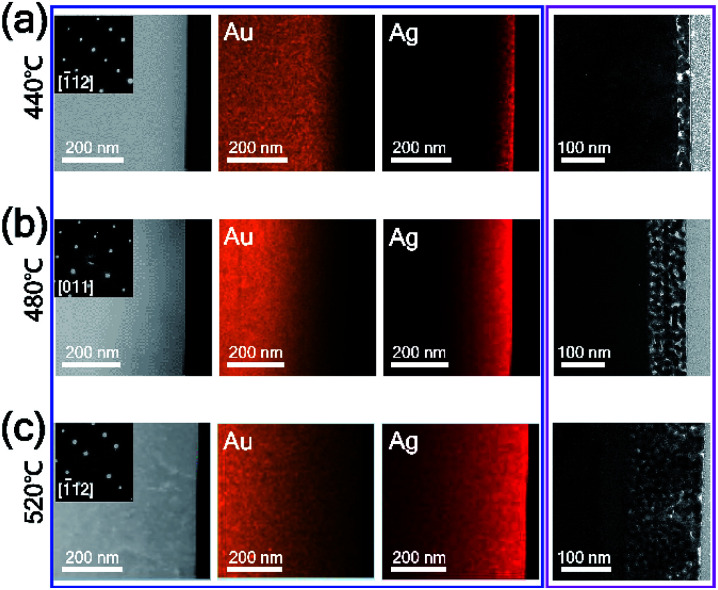
(a–c) Cross-sectional STEM (left panel in blue box) and EDS mapping (middle and right panels in blue box) images of AuAg alloy nanoplates synthesized at temperatures of (a) 440, (b) 480, and (c) 520 °C and cross-sectional TEM images of the corresponding nanoporous Au plates (magenta box). Insets in the STEM images are SAED patterns. In the EDS mapping images, the orange and red colors represent Au and Ag atoms, respectively.

To investigate the reaction temperature-based composition control of AuAg alloy nanoplates and the corresponding control of the intra-nanogap of porous nanoplates, TEM analysis was performed, as shown in Fig. 3. The AuAg alloy nanoplates were synthesized at 440, 480, and 520 °C, and the corresponding porous nanoplates were prepared by chemical etching. First, we obtained scanning transmission electron microscopy (STEM) and energy dispersive X-ray spectroscopy (EDS) mapping images of the AuAg alloy nanoplates (blue box in Fig. 3). At the reaction temperature of 440 °C, Ag atoms were present in the outermost region of the nanoplate. As the reaction temperature increased, Ag atoms were deeply spread into the nanoplates. Notably, the alloy nanoplates provided clean selected area electron diffraction (SAED) patterns, preserving the single-crystalline nature of the nanoplates. The zone axes of the SAED patterns are also written in the insets. The magnified box in Fig. 3 displays the cross-sectional TEM images of the porous Au nanoplates after etching of the alloy nanoplates. As the reaction temperature increased, the porous areas of the nanoplates became wider. At 440 °C, the thickness of the nanoporous region was ∼35 nm. At 480 °C, the thickness was ∼100 nm. At 520 °C, a nanoporous region that was ∼170 nm thick was formed. From the TEM and EDS analysis results, we found an interesting relationship between the composition of Ag and the thickness of the porous region. The porous structures were formed up to the region where the composition of Ag was 50%. This result agrees with a previous report in which the dissolution of Ag in the AuAg alloy is limited when the Au atomic composition is larger than 44%.^45,46^ In this experiment, we synthesized single-crystalline AuAg alloy nanoplates using Au nanoplates and AgI precursors. The composition of alloy nanoplates was controllable by adjusting the reaction temperature. Consequently, we constructed nanoporous plates that controlled the size and thickness of the porous area. Actually, the pore size of nanostructure can be controlled by not only the composition of alloy but also the de-alloying time.^47^ We focused on the relation between the pore size and the composition of alloy, thus we fixed the de-alloying time (*i.e.* etching time) at 2 min.

### Nanogap control

**Fig. 4:**
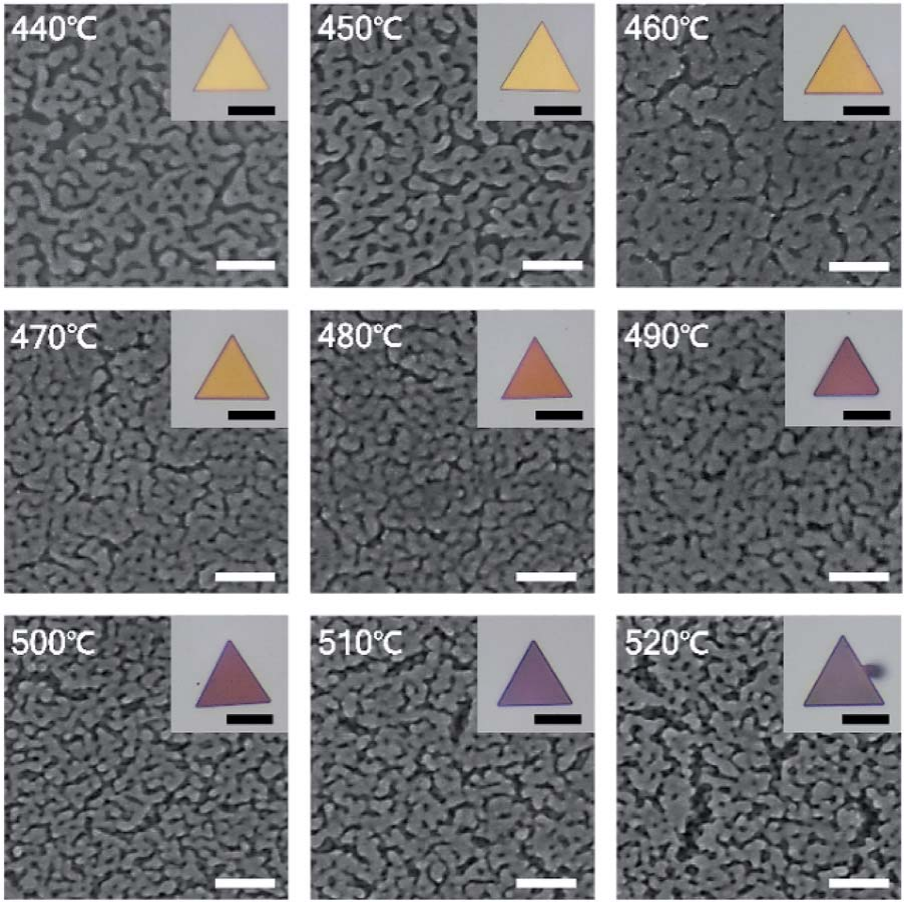
SEM images of nanoporous Au plates synthesized at temperatures ranging from 440 to 520 °C with intervals of 10 °C. Insets are optical images of the corresponding nanoporous Au plates. Scale bars denote 100 nm for SEM images and 20 μm for optical images.

For the precise control of nanoporous plates, we tried to synthesize AuAg alloy nanoplates at reaction temperatures from 440 to 520 °C with intervals of 10 °C. After the preparation of AuAg alloy nanoplates at various temperatures, the composition of Ag on the surfaces of alloy nanoplates was measured using scanning electron microscopy (SEM)-EDS (Fig. S4†). The atomic ratio of Ag increased from 36.9 to 72.6% as the reaction temperature increased from 440 to 520 °C, suggesting that the temperature-based control of alloy nanoplates is feasible. Next, we chemically etched the various AuAg nanoplates to obtain intra-nanogap controllable Au plates. Fig. 4 shows the optical and SEM images of the porous nanoplates. As shown in the optical micrographs (inset of Fig. 4), all porous nanoplates retained triangular shapes regardless of the reaction temperature, but the colors of the porous nanoplates changed from gold to reddish to purple as the temperature increased. This color change could be attributed to the difference in localized surface plasmon resonance.^48,49^ Since the gap size and the thickness of the porous region are different in each of the porous nanoplate samples, their optical properties are varied. The SEM images clearly show the surface morphologies of the nanoporous plates. Numerous nanosized gap structures were formed on every Au plate. We also found that relatively large gap structures were formed at reaction temperatures of 440 and 520 °C. Specifically, the pore size of the nanoplates decreased as the reaction temperature increased from 440 to 480 °C, while it increased as the temperature increased from 480 to 520 °C.

**Fig. 5:**
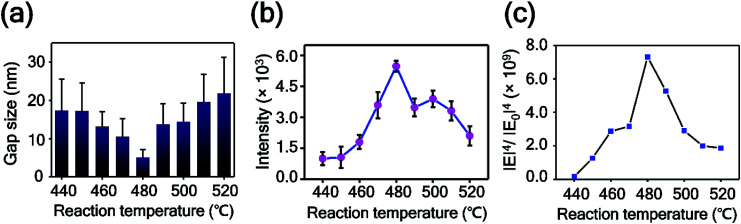
(a) Plot of intra-nanogap sizes of Au plates *versus* the reaction temperature. The data represent the mean plus standard deviation from 40 measurements. (b) Plot of the 1141 cm^−1^ band intensity of 4-ABT *versus* the reaction temperature. The data represent the mean plus and minus standard deviation from 12 measurements. (c) Plot of numerically calculated electrical enhancements of nanoporous Au plates *versus* the reaction temperature.

To prove this observation further, we analyzed the intra-nanogap structures of Au plates quantitatively. Fig. 5a is the plot of the mean surface gap size of Au plates *versus* the reaction temperature. The mean surface gap size and the standard deviation were determined from the 40 randomly selected intra-nanogap regions on each Au plate. The plot corresponds well to the SEM images of Fig. 4. The gap size of the nanoplate decreased from 17.38 ± 8.21 to 5.01 ± 2.15 nm as the reaction temperature increased from 440 to 480 °C, and the gap size increased from 5.01 ± 2.15 to 21.85 ± 9.37 nm as the temperature increased from 480 to 520 °C. At the reaction temperature of 480 °C, the intra-nanogap of the Au plate was the smallest. We estimated that the gap size of the nanoporous Au plate is related to the adsorption of AgI and the thermal diffusion of Ag. According to the proposed synthetic mechanism of AuAg alloy nanoplates, AgI vapor first adsorbs on the Au nanoplate and then decomposes to Ag and I. At the low temperatures of 440 and 450 °C, the decomposed Ag atoms are present at the outermost surface of nanoplates because the thermal energy is insufficient to diffuse Ag atoms deeply into the Au nanoplate. When these AuAg nanoplates are chemically etched, relatively large porous structures are obtained in the outer region of the nanoplates. As the reaction temperature increased to 460, 470, and 480 °C, the Ag atoms diffused into the Au nanoplate more deeply. When these alloy nanoplates are etched, the gap size of the Au plate becomes smaller, and the porous region becomes thicker, depending on the reaction temperature. Therefore, the intra-nanogap size of the Au plate gradually decreases at reaction temperatures from 440 to 480 °C. At the high reaction temperature of over 480 °C, the Ag ratio of the nanoplates increases dramatically because the transported amounts of AgI increase abruptly. When these nanoplates are etched, large and thick porous structures with some cracks are obtained. Therefore, the gap size of the Au plate increases again from the reaction temperatures of 480 to 520 °C. This result indicates that the present method can sophisticatedly manipulate the 3-dimensional porous structures by simply changing the reaction temperature. Compared to the solution phase synthesis,^22,50^ nanoporous structures obtained from this approach have clean surfaces without any ligand molecules because the structures are synthesized based on the single-crystalline Au nanoplates and the AuAg alloying reaction occurs in vapor phase. This porous structures with ligand-free and clean surface provide effective and stable SERS signal enhancement.

### SERS measurement and computational calculation

To examine the SERS enhancements of the intra-nanogap controllable Au plates, we measured the SERS spectra of 4-aminobenzenethiol (4-ABT) from the nanoporous Au plates. For the measurements of SERS, 4-ABT was modified on the nanoporous Au plates through the Au–S bond. Fig. 5b shows the 1141 cm^−1^ band intensity of 4-ABT plotted as a function of the alloying temperature. The corresponding full SERS spectra are displayed in Fig. S5.† The SERS signals increased from the reaction temperatures of 440 to 480 °C and were inversely proportional to the gap size of the nanoporous plates. From the reaction temperatures of 480 to 520 °C, the SERS intensity decreased. As shown in Fig. 5b, the nanoporous Au plate at 480 °C with an intra-nanogap size of 5.01 nm showed the maximum SERS intensity. Considering that the SERS enhancement is highly dependent on the nanosized gap structures and the smaller pore size provides the stronger SERS enhancement,^51^ this result is reasonable. The SERS intensities of the 490–520 °C samples are stronger than those of the 440–470 °C samples, even though the gap sizes of the samples are similar. This result could be explained by the thickness difference in the porous area. Although the intra-nanogap sizes are similar, the 490–520 °C samples have thicker porous regions than the 440–470 °C samples, providing stronger SERS signals. We estimated the EF value of the nanoporous plate prepared at 480 °C from the following equation:^52^EF = (*I*_plate_/*N*_plate_)/(*I*_normal_/*N*_normal_)where *I*_plate_ and *N*_plate_ are the intensity and the number of 4-ABT contributing to the SERS signals, respectively. *I*_normal_ and *N*_normal_ are the intensity and the numbers of 4-ABT contributing to the normal Raman signals, respectively. The normal Raman signals of 4-ABT were obtained from a 0.1 M solution of 4-ABT. *N*_plate_ was calculated from the monolayer coverage of a single 4-ABT (0.39 nm^2^).^53,54^ The diameter of the laser was estimated as 1 μm, and the irradiated depth of the porous structure was estimated as 100 nm, as shown in Fig. 3b. The factor 7 is considered for the calculation of surface area because the total surface area of porous (<13 nm) structure is about 7 times larger than that of the flat structure.^55^ According to the equation, the EF of the optimum nanoporous plate was 3.7 × 10^7^. Detailed calculation of the EF is described on the ESI.†Fig. 5c shows the plot of numerically calculated electrical enhancements for the nanoporous Au plates prepared at 440–520 °C. The result of the computational simulation is consistent with the experimental result in Fig. 5b. We developed intra-nanogap controllable Au plates by investigating the AuAg alloying and etching processes. As a result, the optimized nanoporous Au plate provided strong SERS enhancements, corresponding to the simulation results.

**Fig. 6:**
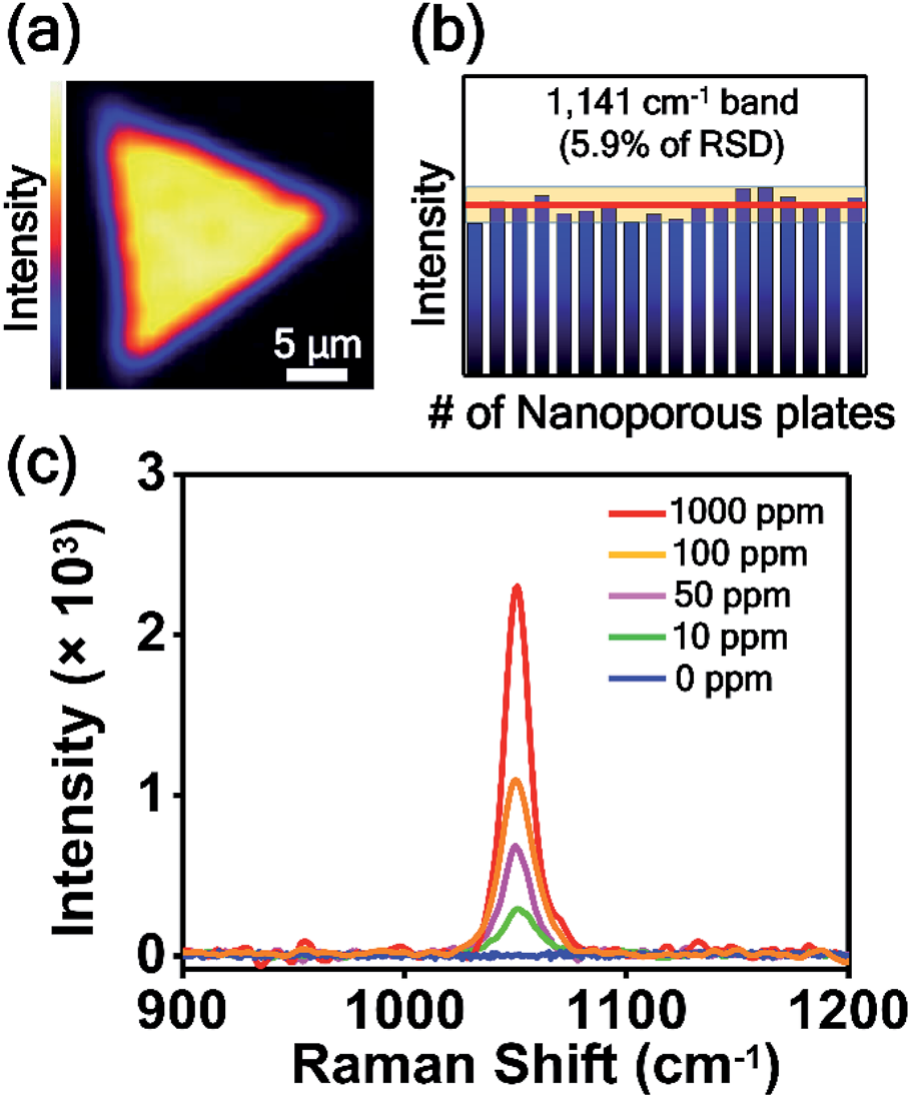
(a) Raman mapping image over the entire area of the nanoporous Au plate. 4-ABT was employed as a Raman molecule, and the brightness in the mapping image was proportional to the 1141 cm^−1^ band intensity. (b) Intensity distribution of the 1141 cm^−1^ band intensity of 4-ABT obtained from 18 replicate samples. The red line represents the average intensity. The yellow zone represents the average intensity ± 5.9% (RSD). (c) SERS spectra of CMIT/MIT measured on nanoporous Au plates depending on the concentration of CMIT/MIT from 0 to 1000 ppm.

For the practical application of SERS-active platforms, it is a priority to develop uniform and reproducible SERS-active nanostructures. We successfully controlled the nanostructured gaps of SERS-active platforms and thus obtained SERS signals with an RSD value of 4.7% from the optimized nanoporous Au plate (Fig. 5b). To further confirm the uniformity of the nanoporous plate, we measured the point-by-point Raman mapping image over the whole area of a nanoporous plate with a step size of 2 μm (Fig. 6a). 4-ABT was employed as a Raman molecule, and the brightness in the mapping image was proportional to the 1141 cm^−1^ band intensity. The uniform brightness over the whole nanoplate reveals that the nanoporous structures were uniformly created and that consistent SERS enhancements were acquired through the whole plate. Furthermore, we tested the reproducibility of the randomly selected 18 nanoporous Au plates. Fig. 6b is the plot of the 1141 cm^−1^ band intensity of 4-ABT *versus* the nanoporous plates. The calculated RSD value was 5.9%. The high uniformity and reproducibility of the nanoporous Au plates are an advancement toward the practical application of SERS for the repeatable and reliable detection of trace molecules.

Finally, the nanoporous Au plate was employed for the detection of toxic chemicals. CMIT/MIT is one of the key chemicals in disinfectants and preservatives in consumer products,^29^ and it was announced as secondary inhalation toxic chemicals by the USA Environmental Protection Agency (EPA). Because the CMIT/MIT molecules have been known to have harmful effects on the bronchi and cause lung injury and even lung cancer,^56–59^ the sensitive detection of CMIT/MIT is highly important. Particularly in South Korea, CMIT/MIT caused serious lung damage to more than 400 people, as those substances were inhaled in a confined area through the humidifiers. This issue has been known as the South Korea humidifier scandal. Since the SERS-based detection of CMIT/MIT is rare, we tried to detect this biocide mixture using nanoporous plates. Fig. 6c shows the SERS spectra of CMIT/MIT obtained from the nanoporous Au plates over a concentration range of 0 to 1000 ppm. The peak at 1050 cm^−1^ increased as the concentration of CMIT/MIT increased, and the detection limit was estimated to be 10 ppm. This detection limit value is noteworthy because the permitted limit of CMIT/MIT is 15 ppm. The signal-to-noise ratio of the SERS spectrum when the CMIT/MIT concentration is 10 ppm is about 30.5, which is calculated by dividing the peak intensity at 1050 cm^−1^ by the standard deviation of intensity of the spectrum where there is no Raman signal.^60^ We anticipate that the intra-nanogap Au plates will be used as SERS sensing platforms for the detection of various kinds of chemical and biological molecules.

## Experimental section

### Material

Au powder (99.99%), AgI (99%), nitric acid (70%), 4-ABT (97%), ethanol (99.5%), and CMIT/MIT were purchased from Sigma Aldrich.

### Synthesis of Au nanoplates

Single-crystalline Au nanoplates were synthesized on a 5 × 5 mm^2^ sapphire substrate in a 1 inch diameter quartz tube using a horizontal hot-wall single zone furnace system.^25^ Briefly, Au powder was placed in the center of the heating zone, and the sapphire substrate was positioned a few centimeters downstream from the Au powder. The Au powder was heated to 1200 °C with a 100 sccm Ar flow rate, and the chamber pressure was maintained at 3 torr. After a reaction time of 3 h, the furnace was cooled to room temperature.

### Synthesis of intra-nanogap Au plates

The as-prepared Au nanoplates were alloyed by a reaction with AgI vapor in a glass reaction tube. AgI powder was placed in the center of the heating zone, and Au nanoplates were positioned a few centimeters downstream from the AgI powder. The glass tube was placed at the center of the heating zone, and the inlet of the glass tube was open to the opposite direction of the carrier gas moving direction. Ar carrier gas flowed at a rate of 150 sccm to transport the vaporized AgI, while the chamber pressure was maintained at 0.7 torr. After a reaction time of 40 min, the furnace was completely cooled to room temperature. Next, nanoporous plates were prepared by selectively etching Ag atoms from the AuAg alloy nanoplates. All of the AuAg alloy plates were immersed in nitric acid for 2 min, washed with distilled water, and dried with N_2_.

### SERS measurements

All of the nanoporous plates, which were prepared at different reaction temperatures, were incubated in a 10^−5^ M 4-ABT solution in ethanol for 12 h. Next, the nanoporous plates were washed several times with pure ethanol and purged with N_2_. The point-by-point Raman mapping image was obtained from a nanoporous plate prepared at 480 °C.

### Computational simulation

The electric field enhancement and the ratio of the intensity of the concentrated field to that of the incident field were calculated by the finite-differential time-domain (FDTD) method. To mimic the nanoporous structures, the diameter of randomly distributed Au spheres and the filling ratio were determined based on the SEM images in Fig. 4. The layer of the randomly distributed spheres was located on the Au substrate. The thickness of the layer changes from 30 to 150 nm according to the cross-sectional TEM images (Fig. 3). By averaging the electric field enhancements at the pores larger than the calculation grid size (2 nm) in the FDTD simulation, the artifacts in the analysis can be avoided. A linearly polarized plane wave with a wavelength of 633 nm is normally incident to the porous layer.

### Detection of CMIT/MIT

For the detection of CMIT/MIT using the nanoporous plates, the concentrated CMIT/MIT solution was diluted with ultrapure water to the desired concentration, and a drop of the CMIT/MIT solution was pipetted out onto the nanoporous plates. SERS spectra were obtained after the droplet was completely dried.

### Instrumentation

SERS spectra and Raman mapping images were obtained using a high-resolution dispersive Raman microscope (ARAMIS, Horiba Jobin Yvon, France). The 633 nm laser with a power of 5 mW was focused on the samples with a beam diameter of 1 μm through a 100× objective. Field-emission SEM images were collected with a Nova230 (FEI). In the SEM observations, samples were coated with Pt to prevent charging effects. TEM images, EDS analysis, and SAED patterns were obtained with a Tecnai G2 F30 S-Twin (FEI) operated at 300 kV. Cross-sectional TEM samples were prepared with an FIB instrumentation (Helios Nanolab 450 F1). XPS spectra were obtained using a Thermo VG Scientific K-alpha instrument.

## Conclusions

We report the synthesis of an intra-nanogap controllable Au plate with uniform and reproducible SERS activity and the application of a nanoporous Au plate to the sensing of trace amounts of a detrimental chemical. The single-crystalline Au nanoplates with ultraflat and ultraclean surfaces were alloyed under a vapor phase reaction with AgI, and the nanoporous Au plates were obtained after the simple etching of the alloy nanoplates. From the analyses of XPS and TEM, we found that AgI selectively adsorbed onto the Au nanoplates and subsequently decomposed to Ag and I. By investigating the alloy formation mechanism, the size and morphology of intra-nanogaps were able to be fine-tuned. The nanoporous Au plates with optimized intra-nanogaps showed excellent uniformity and reproducibility for SERS signals. Furthermore, the nanoporous Au plate was applied to the label-free detection of a harmful biocide mixture (CMIT/MIT) and successfully detected CMIT/MIT at a low concentration of 10 ppm. We anticipate that the intra-nanogap controllable Au nanoplates will be of great significance for the practical SERS-based sensing of chemical and biological analytes.

## Conflicts of interest

There are no conflicts to declare.

## Supplementary Material

RA-009-C9RA01813A-s001
