# Supplementary material for: Intra-nanogap controllable Au plates as efficient, robust, and reproducible surface-enhanced Raman scattering-active platforms
Source: RSC Adv. 2019 Apr 29;9(23):13007–15. doi: 10.1039/c9ra01813a (PMC9063784; doi:10.1039/c9ra01813a)
Supplement: RA-009-C9RA01813A-s001 [file RA-009-C9RA01813A-s001.pdf]

Electronic Supplementary Material (ESI) for RSC Advances.
This journal is © The Royal Society of Chemistry 2019

Supplementary information

Intra-Nanogap Controllable Au Plates as Efficient, Robust, and Reproducible Surface-Enhanced Raman Scattering-Active Platforms

Siyeong Yang,‡[a] Minjin Kim,‡[a] Sanghyeok Park,[b] Hongki Kim,[c] Jinyoung Jeong,[d,e] Juyeon Jung, [c,d] Eun-Kyung Lim,[c,d] Min-Kyo Seo,[b] Bongsoo Kim,*[a] and Taejoon Kang*[c,d]

[a]Department of Chemistry, KAIST, 291 Daehak-ro, Yuseong-gu, Daejeon 34141, Korea.

[b]Department of Physics, KAIST, 291 Daehak-ro, Yuseong-gu, Daejeon 34141, Korea.

[c]Bionanotechnology Research Center, 125 Gwahak-ro, Yuseong-gu, KRIBB, Daejeon 34141, Korea.

[d]Department of Nanobiotechnology, KRIBB School of Biotechnology, UST, 217 Gajeong-ro, Yuseong-gu, Daejeon 34113, Korea.

[e]Environmental Disease Research Center, 125 Gwahak-ro, Yuseong-gu, KRIBB, Daejeon 34141, Korea.

‡These authors contributed equally to this work.

Corresponding Author

*E-mail: bongsoo@kaist.ac.kr (B.K.); kangtaejoon@kribb.re.kr (T.K.)

The enhancement factor (EF) of the porous plate was estimated using the equation:[1]

$$EF = (I_{plate}/N_{plate})/(I_{normal}/N_{normal})$$

where I_{plate} and N_{plate} are the intensity and the number of 4-ABT contributing to the SERS signals, respectively. I_{normal} and N_{normal} are the intensity and the numbers of 4-ABT contributing to the normal Raman signals, respectively.

As the peak intensity at 1,141 cm^{-1} for 4-ABT, I_{plate} and I_{normal} are 5270 and 24, respectively. N_{plate} and N_{normal} are the number of adsorbed 4-ABT on the porous plate and Si substrate within the laser spot size, respectively, and estimated as follows:[2]

$$N_{plate} = 7 \times (2 \times A/2 \times \pi \times d) / (0.39 \times 10^{-18}) = 56.1 \times 10^{3}$$

$$N_{normal} = 5 \times 10^{-6} \times 0.1 \times 6.02 \times 10^{23} \times \pi \times (A/2)^{2}/s^{2} = 0.945 \times 10^{10}$$

where A is the laser spot size (1 μm), d is the thickness of porous region (100 nm), and s is the edge length of the Si substrate (5 mm). Finally, the estimated EF value was 3.7×10^{7}.

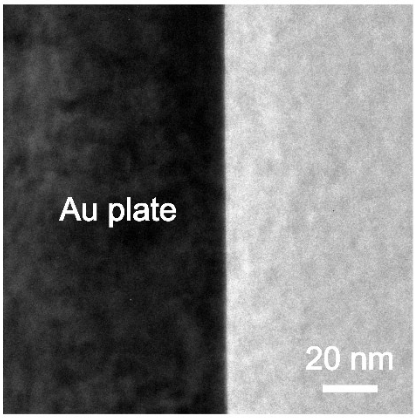

Fig. S1 Cross-sectional TEM image of the Au nanoplate.

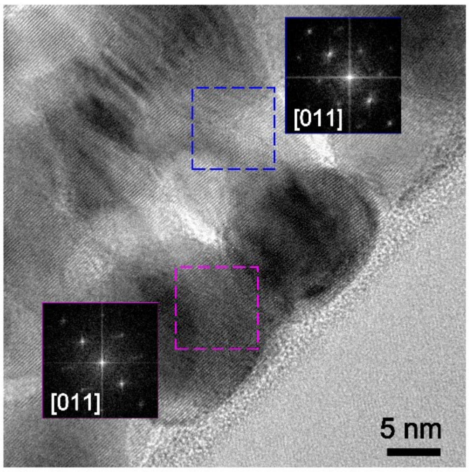

Fig. S2 HR-TEM image of the nanoporous Au plate. Insets are FFT patterns of dashed line boxes.

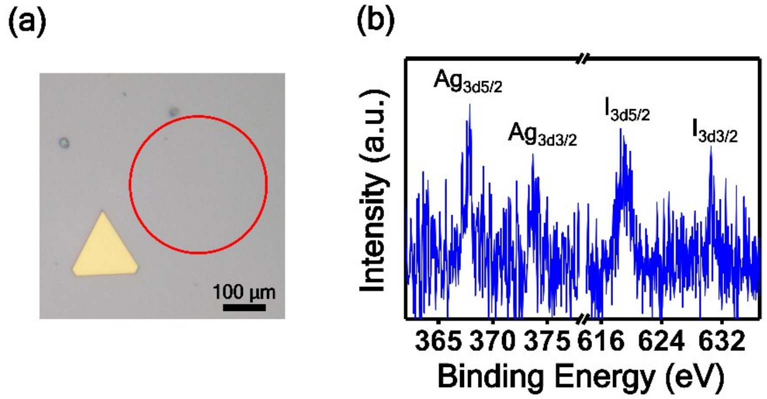

Fig. S3 (a) Optical image of the AuAg alloy nanoplate on a substrate. (b) XPS spectra obtained from the red circle in (a).

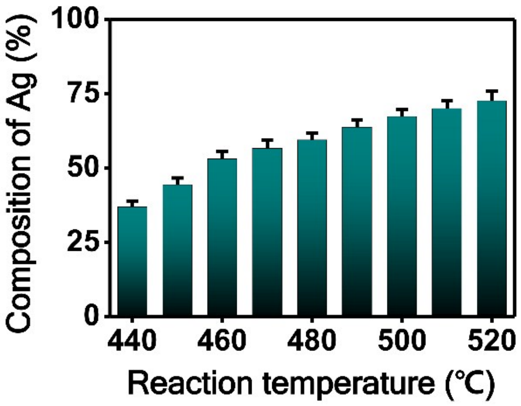

Fig. S4 Composition of Ag on the surfaces of AuAg alloy nanoplates synthesized at temperatures ranging from 440 to 520°C with intervals of 10°C. The atomic ratio of Ag increases from 36.9 to 72.6% as the reaction temperature increases from 440 to 520°C. Data represent the mean plus standard deviation from twelve measurements.

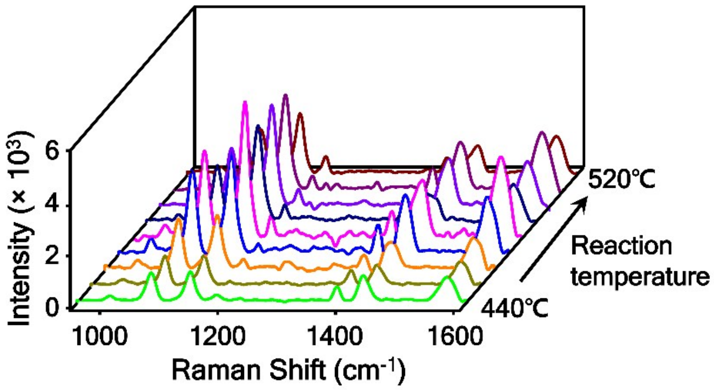

Fig. S5 Full SERS spectra of 4-ABT measured on nanoporous Au plates depending on the reaction temperatures from 440 to 520°C.

Reference

1. G. Eom, H. Kim, A. Hwang, H. Y. Son, Y. Choi, J. Moon, D. Kim, M. Lee, E. K. Lim and J. Jeong, *Advanced Functional Materials*, 2017, **27**, 1701832.
2. J. Qi, P. Motwani, M. Gheewala, C. Brennan, J. C. Wolfe and W. C. Shih, *Nanoscale*, 2013, **5**, 4105–4109.
